# In Vitro and Biological Evaluation of Oral Fast-Disintegrating Films Containing Ranitidine HCl and Syloid^®^ 244FP-Based Ternary Solid Dispersion of Flurbiprofen

**DOI:** 10.3390/pharmaceutics16020164

**Published:** 2024-01-24

**Authors:** Aisha Rashid, Muhammad Irfan, Yousaf Kamal, Sajid Asghar, Syed Haroon Khalid, Ghulam Hussain, Abdulrahman Alshammari, Thamer H. Albekairi, Metab Alharbi, Hafeez Ullah Khan, Zunera Chauhdary, Thierry F. Vandamme, Ikram Ullah Khan

**Affiliations:** 1 Department of Pharmaceutics, Faculty of Pharmaceutical Sciences, Government College University Faisalabad, Faisalabad 38000, Pakistan; draisharashid@gmail.com (A.R.); manipharma1@gmail.com (M.I.); sajuhappa@gmail.com (S.A.); haroonkhalid80@gmail.com (S.H.K.); 2 Hamdard Institute of Pharmaceutical Sciences, Hamdard University Karachi, Islamabad Campus, Islamabad 45550, Pakistan; yousafpharmacist1@gmail.com; 3 Department of Physiology, Faculty of Life Sciences, Government College University Faisalabad, Faisalabad 38000, Pakistan; hussain806@gmail.com; 4 Department of Pharmacology and Toxicology, College of Pharmacy, King Saud University, P.O. Box 2455, Riyadh 11451, Saudi Arabia; abdalshammari@ksu.edu.sa (A.A.); thalbekairi@ksu.edu.sa (T.H.A.); mesalharbi@ksu.edu.sa (M.A.); 5 Department of Pharmaceutics, College of Pharmacy, University of Sargodha, Sargodha 40100, Pakistan; qarani_pharmacist@yahoo.com; 6 Department of Pharmacology, Faculty of Pharmaceutical Sciences, Government College University Faisalabad, Faisalabad 38000, Pakistan; zunerach@yahoo.com; 7 Centre de Recherche en Biomédecine de Strasbourg (CRBS), Inserm/Unistra, UMR 1260 Regenerative NanoMedecine, Université de Strasbourg, 1 Rue Eugène Boeckel, 67000 Strasbourg, France; vandamme@unistra.fr

**Keywords:** HPMC E5, Syloid^®^ 244FP, poloxamer^®^ 188, solid dispersion, composite films, flurbiprofen, ranitidine hydrochloride

## Abstract

Flurbiprofen (FBP), a nonsteroidal anti-inflammatory drug (NSAID), is commonly used to treat the pain of rheumatoid arthritis, but in prolonged use it causes gastric irritation and ulcer. To avoid these adverse events of NSAIDs, the simultaneous administration of H_2_ receptor antagonists such as ranitidine hydrochloride (RHCl) is obligatory. Here, we developed composite oral fast-disintegrating films (ODFs) containing FBP along with RHCl to provide a gastroprotective effect as well as to enhance the solubility and bioavailability of FBP. The ternary solid dispersion (TSD) of FBP was fabricated with Syloid^®^ 244FP and poloxamer^®^ 188 using the solvent evaporation technique. The synthesized FBP-TSD (coded as TSD) was loaded alone (S1) and in combination with plain RHCl (S2) in the composite ODFs based on hydroxypropyl methyl cellulose E5 (HPMC E5). The synthesized composite ODFs were evaluated by in vitro (thickness, folding endurance, tensile strength, disintegration, SEM, FTIR, XRD and release study) and in vivo (analgesic, anti-inflammatory activity, pro-inflammatory cytokines and gastroprotective assay) studies. The in vitro characterization revealed that TSD preserved its integrity and was effectively loaded in S1 and S2 with optimal compatibility. The films were durable and flexible with a disintegration time ≈15 s. The release profile at pH 6.8 showed that the solid dispersion of FBP improved the drug solubility and release when compared with pure FBP. After in vitro studies, it was observed that the analgesic and anti-inflammatory activity of S2 was higher than that of pure FBP and other synthesized formulations (TSD and S1). Similarly, the level of cytokines (TNF-α and IL-6) was also markedly reduced by S2. Furthermore, a gastroprotective assay confirmed that S2 has a higher safety profile in comparison to pure FBP and other synthesized formulations (TSD and S1). Thus, composite ODF (S2) can effectively enhance the FBP solubility and its therapeutic efficacy, along with its gastroprotective effect.

## 1. Introduction

Rheumatoid arthritis (RA) is a complex autoimmune and inflammatory disease. It mainly attacks the joints, and usually many joints at once. The signs and symptoms of RA include inflammation, stiffness, severe pain, muscle weakness and reduced joint motion. Long term consequences can include the destruction of the synovial joint and physical disability [1]. According to the literature, RA affects 0.5 to 1% of the worldwide population [2]. It affects both genders at any age, but data revealed that females are three times more prone to RA than males [3]. The first line treatment to manage RA symptoms is NSAIDs. However, the prolonged use of these drugs cause gastrointestinal (GI) bleeding or stomach ulcer [1]. Therefore, due to the higher incidence of NSAID-related adverse reactions, H_2_ receptor antagonist drugs are a promising supportive treatment in such situations [4]. The H_2_ receptor antagonist reduces the stomach acid and ultimately lowers the risk of GI irritation and bleeding in patients taking NSAIDs. Therefore, their combination in a single formulation might prove more economical and convenient for geriatric patients.

FBP is a potent NSAID which is frequently used to treat pain and inflammation associated with arthritis. However, when taken orally, it presents unwanted effects such as GI irritation, GI bleeding, ulcer, and other systemic effects. Moreover, it exhibits poor aqueous solubility as it belongs to BCS class II [5]. All these factors limit its therapeutic efficacy. On the other hand, RHCl is a H_2_ receptor antagonist that inhibits gastric acid secretions [6]. It has short half-life, i.e., 2.2 h, and shows low absorption on oral administration as it belongs to BCS class III. Moreover, it also undergoes hepatic metabolism [7]. Therefore, both drugs exhibit low bioavailability. Composite ODFs are usually developed at stamp size from hydrophilic polymers, which offers several advantages when compared to other oral solid dosage forms: they are easy to swallow, rapidly disintegrate without water intake, have a rapid onset of action, give tolerable mouthfeel, show improved patient compliance, especially for geriatric and pediatric patients, avoid the degradation of active pharmaceutical ingredients susceptible to degradation in the stomach or intestine, and lastly are cost effective as well. Moreover, the size and thickness of the ODF can be adjusted to meet the individual dosage requirements of geriatric patients [8]. Visser, Dohmen [9] and Alhayali, Vuddanda [8] reported that hydroxypropyl methyl cellulose (HPMC) forms good films and has optimal physicomechanical properties compared to other tested excipients. Łyszczarz, Hofmanová [10] successfully developed ODFs containing a poloxamer^®^ 407-based solid dispersion of aripiprazole. In another study, Senta-Loys, Bourgeois [11] fabricated an ODF based on the solid dispersion of tetrabenazine. The solid dispersion (SD) method is the oldest and most widely used solubilization technique. SD production has different approaches, such as solvent evaporation and melt cooling, which were later updated to be better suited for commercialization. Solvent evaporation methods include freeze-drying, supercritical fluids, nitrogen steam and rotary evaporation. Melting methods include traditional methods (solution and suspension methods) and optimized methods (hot-stage extrusion, metrex™ and melt agglomeration) [12,13]. In this study, the traditional solvent evaporation approach was used as it is easy to perform, provides the maximum yield of final product and has been reported to have high drug entrapment efficiency. A sublingual bioadhesive film containing a solid dispersion of furosemide was fabricated to enhance the bioavailability of the encapsulated drug [14]. Syloid^®^ 244FP is a non-order mesoporous silica which is frequently used as a carrier for solubility enhancement. It is reported that the large pore volume and surface area offers an efficient adsorption of drugs. Moreover, the large number of silicon hydroxyl groups on its surface develop hydrogen bonding with drugs, which subsequently improves the drug release profile [15,16,17]. Poloxamer^®^ 188 is a nonionic, linear, amphiphilic block copolymer composed of hydrophilic polyoxyethylene and hydrophobic polyoxypropylene, with excellent solubilizing capacity. Furthermore, the presence or coating of poloxamer^®^ 188 also provides a gastroprotective effect [18] and significantly improves the intestinal absorption of the drug [19]. As per the literature, poloxamer^®^ 188 is frequently used as a hydrophilic carrier along other carriers for the development of TSD [20,21,22,23,24].

The novelty and uniqueness of the current research lies in the co-loading of plain RHCl and solid dispersion of FBP in ODF for the very first time. Later, their efficacy was evaluated by various in vitro and in vivo tests. Therefore, the aim was to prepare a combinative product of FBP and RHCl to enhance bioavailability and gastro protection and possibly improve compliance in geriatric patients.

## 2. Material and Methods

### 2.1. Material

In a composite ODF film, hydroxypropyl methyl cellulose E5 (HPMC E5, Alfa Aesra, Karlsruhe, Germany) was used as a film matrix, and propylene glycol (PG, Daejung Chemicals and Metals Co., Ltd., Siheung, Republic of Korea) was used as a plasticizer. Superdisintegrant pearlitol flash^®^ was received as a gift from Roquette (Lestrem, France). Flurbiprofen (FBP) and ranitidine HCl (RHCl) were kindly provided by Axis Pharmaceuticals (Faisalabad, Pakistan). The ternary solid dispersion (TSD) of FBP was prepared with Syloid^®^ 244FP EU (Grace GmbH, Worms, Germany) and poloxamer^®^ 188 (Sigma-Aldrich, Darmstadt, Germany), whereas dichloromethane (DCM, Icon Chemical, Schlüchtern, Germany) was used as the solvent. All other excipients used were of analytical grade.

### 2.2. Methods

#### 2.2.1. Development of Solid Dispersion

**Figure 1:**
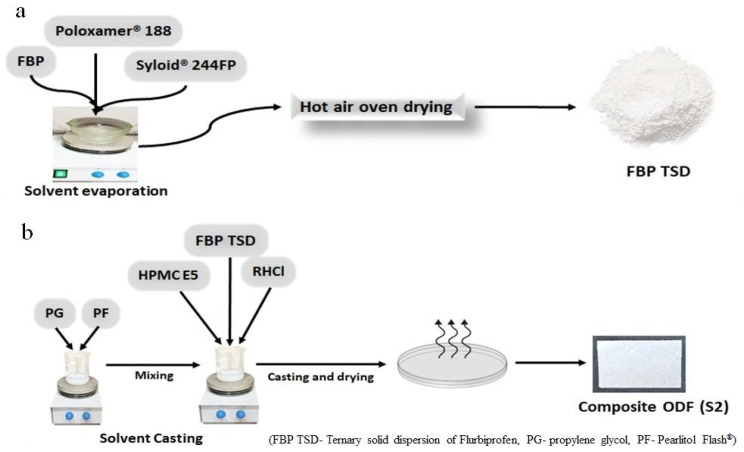
Synthesis of ternary solid dispersions (**a**); synthesis of composite ODFs (**b**).

Binary and ternary solid dispersions were prepared by the solvent evaporation method [25]. For BSD, FBP (0.5 g) was dissolved in DCM (50 mL). This solution was taken in a glass mortar, and then Syloid^®^ 244FP was added in parts to this solution while stirring was continued for 30 min at 300 rpm. After that, the DCM was evaporated in an oven at 40 °C for 3 h. The solid product was triturated and sieved through 40 mesh. The sieved powder was stored in airtight glass vials and placed in a desiccator until further analysis. Similarly, TSD was prepared with a little modification (Figure 1a). After dissolving FBP (0.5 g) in DCM (50 mL), poloxamer^®^ 188 (0.25 g) was added to it with constant stirring at 300 rpm. When poloxamer^®^ 188 was completely dissolved, then specific amount of Syloid^®^ 244FP (0.5 g) was added in parts to this solution and stirred for 30 min at 300 rpm. Finally, the dried product was sieved and stored, until further analysis.

#### 2.2.2. Development of Composite ODFs

The composite film (S1) was prepared by solvent casting method [26], as follows. Initially, 0.3 g HPMC E5 was dissolved in 5 mL distilled water, then 0.15 g of prepared TSD was added (Table 1). PG (10% *w*/*w* of polymer) and Pearlitol Flash^®^ (10% *w*/*w* of polymer) were dissolved in 5 mL distilled water. This solution was blended with HPMC E5 solution and cast in a Petri dish and dried over night at room temperature. The dried film was wrapped in aluminum foil and stored in a desiccator until further analysis. Likewise, dual-drug-loaded composite film (S2) was prepared with little modification; i.e., first, RHCl (0.075 g) was dissolved in polymer solution, and after that, a designated amount of TSD was added to the solution (Figure 1b). A blank film (S0) was also prepared in a similar way but without drugs.

### 2.3. Characterization of TSD and Composite ODFs

#### 2.3.1. Micromeritic Properties of TSD

##### Powder Density

Powder density is used to evaluate the packing properties of powder into capsules but may also affect several pharmaceutical processes like flow, mixing and tableting. Bulk density was measured by introducing an accurate amount of solid dispersion into a 10 mL graduated cylinder, and the powder was carefully leveled without compacting it. The apparent untapped volume was noted. After that, the cylinder was tapped carefully and the volume was noted. Tapping was stopped when no change in volume was observed. The bulk density and tapped bulk density were calculated by the following equations [15].

$$Bulk\;Density=\frac{Weight\;of\;powder}{Volume\;of\;powder}\;Tapped\;Bulk\;Density=\frac{Weight\;of\;powder}{Volume\;of\;powder\;after\;100\;tapings}$$

##### Compressibility Index (Ci)

This is an indirect measurement of bulk density, surface area, size, shape, moisture content, and the cohesiveness of materials since all of them can influence the compressibility index or Carr’s Index. It was calculated as follows [15]:

$$Ci(\%)=\frac{Tapped\;density-bulk\;density}{tapped\;density}\times 100$$

whereas a Ci value 0–10% shows excellent flow characteristics and above 25% reflects poor flow characteristics.

##### Hausner’s Ratio

Hausner’s ratio is another index for calculating the flowability of powder and was calculated as follows [15]:

$${Hausner}^{’}s\;Ratio=\frac{Tapped\;density}{Bulk\;density}$$

A value less than 1.2 is preferable for free flow. However, a Hausner’s ratio between 1 and 1.1 specifies excellent flow properties.

##### Angle of Repose

The angle of repose was calculated by passing powder through the funnel on a horizontal surface. The height (h) of the heap of powder and radius (r) of the cone base were measured. The angle of repose (θ) was calculated by the following equation [15]:

$$Tan\theta =\frac{h}{r}$$

An angle less than 40° reflects free flow properties, while an angle between 25 and 30° specifies excellent flow properties.

#### 2.3.2. Solubility Study of TSD

The solubilities of FBP and TSD were determined in distilled water, a USP phosphate buffer of pH 6.8 and a USP hydrochloric acid buffer of pH 1.2. The excess amount of drug or solid dispersion was added in a falcon tube containing 3 mL of respective solvent. It was placed in a thermostatic shaking water bath at 37 °C and agitated at 100 rpm for 72 h. Then, the samples were centrifuged for 30 min at 6000 rpm. The obtained clear supernatant was passed through a 0.45 µm nylon syringe filter (HyDocs, London, UK), diluted and assayed using a UV–Visible Spectrophotometer (CECIL CE7400S, Cambridge, UK) at λ = 247 nm to determine the drug dissolved per mL [10].

#### 2.3.3. Drug Content in TSD

Briefly, 30 mg of drug-loaded TSD was first dispersed in 10 mL of DCM and later diluted with phosphate buffer (pH 6.8). DCM was removed by the vigorous agitation of the solution for 1 h and passed through a 0.45µm nylon syringe filter (HyDocs, London, UK). After filtration, the absorbance of FBP was measured spectrophotometrically at 247 nm. The results were expressed in % drug content [27]:

$$\%\;drug\;content=\frac{Actual\;amount\;of\;drug\;in\;TSD}{Theoratical\;amount\;of\;drug\;in\;TSD}\times 100$$

#### 2.3.4. Physical Parameters of Composite ODFs

##### Thickness

The thickness of the film was measured by using an electronic digital micrometer (5202-100, SHAHE, Liushi, China) with an accuracy of 0.001 mm. Thickness was measured from the center and edges of the film, and the mean thickness was reported [10].

##### In Vitro Disintegration Time

The in vitro disintegration time of the film was measured by adopting a visual method. The 6 cm^2^ film strip was placed in a Petri dish with 10 mL phosphate-buffered solution (PBS) of 6.8 pH. Disintegration time was considered when the film completely disintegrated into components, and the mean was reported [28].

#### 2.3.5. Mechanical Parameters of Composite ODFs

##### Folding Endurance

Folding endurance was measured by repeatedly folding the film at the same place until the film breaks. The number of the times the film folded without breaking was taken as the folding endurance value [29]. The mean value of triplicate observation was reported.

##### Tensile Strength

Tensile strength was measured by using a universal testing machine (INSTRON 3366-10 KN, Instron^®^ GmbH, Darmstadt, Germany) equipped with a 10 KN loaded cell. The film strip (5 × 2 cm^2^) was clipped with a clamp with one side fixed and the other side moveable. Both clamps were positioned at a distance of 3 mm. The film was pulled by the upper clamp at the rate of 5 mm min^−1^ until it broke, to determine the tensile strength [10].

#### 2.3.6. Drug Content of Composite ODFs

ODF of 6 cm^2^ was completely dissolved in ethanol and diluted with phosphate buffer (pH 6.8). The contents of FBP and RHCl were determined by using a UV/Vis Spectrophotometer (CECIL CE7400S, Cambridge, UK) at 247 nm and 315 nm, respectively [29].

#### 2.3.7. Solid State Characterization of TSD and Composite ODFs

##### Scanning Electron Microscopy (SEM)

Microstructural analyses of the surface of pure drug (FBP and RHCl), TSD, and the films (S0, S1 and S2) were carried out by using SEM (Cube series, EMCRAFTS, Sungdong-gu, Seoul, Republic of Korea). The sample was placed on an aluminum stub using double-sided adhesive tape and coated with gold under vacuum before observation [30].

##### Fourier Transform Infrared Spectroscopy (FTIR)

To evaluate the drug–polymer compatibility, FTIR spectra were obtained using Nicolet 6700 FTIR spectrometers (Thermo Electron, Waltham, MA, USA) in the spectral region of 4000–500 cm^−1^ [30].

##### X-ray Diffractometry (XRD)

XRD was carried out to investigate the effect of TSD and the ODF formulation on the crystallinity of the drug. The sample was irradiated with an X-Ray diffractometer (D8 ADVANCE, Bruker, Karlsruhe, Germany) with monochromatized X-rays (Cu-kα), which was operated at 30 mA and 30 kV. The data were analyzed at a scanning rate of 2° min^−1^, over the 5–60° diffraction angle (2θ) range at a step size of 0.02°. The XRD patterns of polymers, drug powder, TSD, and the films were recorded [31].

### 2.4. In Vitro Drug Release Profile of TSD and Composite ODFs

The in vitro drug release of each formulation, TSD, S1 and S2, was evaluated using the dialysis membrane method [32]. For FBP release study, each formulation was added to a pre-activated cellulose acetate membrane pouch (Spectra/Por^®^ dialysis membrane, MWCO 10,000 Da) separately. Then, 10 mL of dissolution media was added to the membrane pouch and sealed. The pouch was exposed to two different dissolution media with pH 6.8 and pH 1.2 in a beaker (receptor compartment) placed in a thermostatic shaking water bath at 37 ± 2 °C under continuous shaking (50 rpm) for 120 min. The receptor compartment contained 200 mL of dissolution media to maintain sink conditions. Then, 5 mL of aliquot was drawn out at predetermined intervals and the same volume was replaced. The concentration of FBP was calculated by using a previously constructed calibration curve, while the release of RHCl from S2 was evaluated in 6.8 pH buffer using the above-mentioned dialysis membrane method. All the experiments were performed in triplicate, and the average values were reported.

### 2.5. In Vivo Study Protocols

In vivo studies were conducted on Wistar rats (150–250 g) and were obtained from an in-house animal facility. The Institutional Review Committee, Government College University Faisalabad, Pakistan, approved all the protocols (Ref. No., GCUF/ERC/17, dated: 3 December 2021). Rats were housed in a controlled environment, i.e., 25 ± 1 °C, relative humidity of 60% ± 10% and appropriate light and dark cycles. All the animals were given standard food and water ad libitum. The amount of S1 and S2 administered were equivalent to 5 mg/kg of FBP. The pure FBP was also administered at 5 mg/kg for comparison [33].

#### 2.5.1. In Vivo Analgesic Activity

The in vivo analgesic effect of pure FBP and synthesized formulations (TSD, S1 and S2) was measured by the hot plate method, originally developed by MacDonald, Woolfe [34]. Animals were divided into 5 groups; each animal was placed on a hot plate at 52 ± 1 °C, and latency time was noted at “0” h. Afterward, the first group served as the control group and was given oral 0.5% carboxy methyl cellulose (CMC) solution; the second and third groups received oral suspension of pure FBP and TSD in 0.5% CMC solution, respectively, whereas the fourth and fifth groups received composite ODFs S1 and S2, respectively. The latency time was measured in seconds after every 30 min interval until 5.5 h. The time to withdraw a hind paw from the surface of hot plate, licking of hind paw or jumping off to avoid heat nociception is called latency time or reaction time. The cut off latency was set to 15 s to prevent tissue damage. The percentage maximum possible analgesia (MPA %) of each group was calculated as follows [35]:

$$MPA\%=\frac{Test\;latency-Control\;latency}{Cut\;off\;time-Control\;latency}\times 100$$

#### 2.5.2. In Vivo Anti-Inflammatory Activity

The paw edema model was employed to investigate the in vivo anti-inflammatory activity of synthesized formulations. Acute edema was induced by injecting 0.1 mL of freshly prepared 1% solution of carrageenan in sub-planter tissues of the left hind paw. Before 30 min of induction, the control group was given 0.5% CMC solution orally; the 2nd and 3rd groups were administered a dose of pure FBP and TSD, respectively. The dose of FBP was 5 mg/kg for pure and TSD, while the 4th and 5th groups were administered composite ODFs, i.e., F1 and F2, respectively. The paw diameter was measured with digital Vernier caliper at 0, 1, 2, 3, 4, 5 and 6 h. The percentage inhibition of edema was calculated using the following equation [33].

$$\%inhibition\;of\;paw\;edema=\frac{\left(Ct-C0\right)\;control-\left(Ct-C0\right)\;treated}{\left(Ct-C0\right)\;control}\times 100$$

where Ct = left hind paw thickness (mm) at time t, C0 = left hind paw thickness (mm) before carrageenan injection, (Ct – C0) control = increase in paw size after carrageenan injection to control rats at time t and (Ct – C0) treated = increase in paw size after carrageenan injection to treated rats at time t.

##### Detection of Pro-Inflammatory Cytokines

For the quantification of TNF-α and IL-6 in rat serum, the blood samples were collected by cardiac puncture from anesthetized rat. Enzyme-linked immunosorbent assay (ELISA) was performed by using commercial kits (Elabscience^®^, Wuhan, China) according to the manufacturer’s instructions [36].

#### 2.5.3. Assessment of Gastroprotective Effect

Wistar rats weighing 160–220 g of either sex were divided into 5 groups (*n* = 3) to compare the ulcerogenic potential of pure FBP and synthesized formulations TSD, S1 and S2. All animals were fasted over night with free supply of water. Rats received 5 mg/kg of pure drug or formulation containing an equivalent quantity of drugs. Treatment was continued for five days, and animals were sacrificed on the 5th day after dosing.

##### Gastric Lesion Index (GLI)

The isolated stomachs of rats were incised through great curvature and washed with normal saline. After the macroscopic examination of gastric tissues, ulcerative lesions were measured by Vernier caliper. An arbitrary score (AS) was given to ulcers: “0” was given to no ulcer/lesion, “0.5” was given to one or more ulcers of length ˂1 mm, “1” was given to ulcers/lesions of length 1–2 mm and “2” was given to ulcers/lesions with length >2 mm. This arbitrary score was multiplied with the number of lesions to find the GLI [37].

$$Gastric\;lesion\;index\;\left(GLI\right)=AS\times No.\;of\;lesion/ulcers$$

After that, the stomachs were then preserved in 10% formalin solution for histopathological analysis.

##### Histopathology

The histopathology of stomach tissues was observed by hematoxylin and eosin (H & E) staining. The stomachs of animals from each group were fixed with 4% paraformaldehyde and embedded in paraffin. Thin sections of 5µm were sliced, stained by H & E staining and observed under an accu-scope 3000-LED microscope [37].

## 3. Results and Discussion

Here, we have attempted to develop composites (ODFs) co-loaded with plain RHCl and TSD of FBP so as to overcome the limitations of FBP and RHCl. We believe that the developed system is a promising contender for multiple reasons: First, the co-loading of RHCl (H_2_ receptor antagonist) with FBP can provide therapeutic effects (treating FBP-induced GI effects). Second, it will bypass GI and the hepatic metabolism of RHCl. Third, solid drug dispersion as well as ODF itself enhances drug FBP solubility and permeability and ultimately improves bioavailability. Fourth, the TSD of FBP provides intestinal drug release. Fifth, from a manufacturing perspective, it is easy to prepare and economical to develop composite ODFs. Sixth, there was an ease of compliance for geriatric patients suffering from rheumatoid arthritis (due to lower administration frequency) and patients having swallowing issues.

### 3.1. Evaluation of Binary and Ternary Solid Dispersions

**Figure 2:**
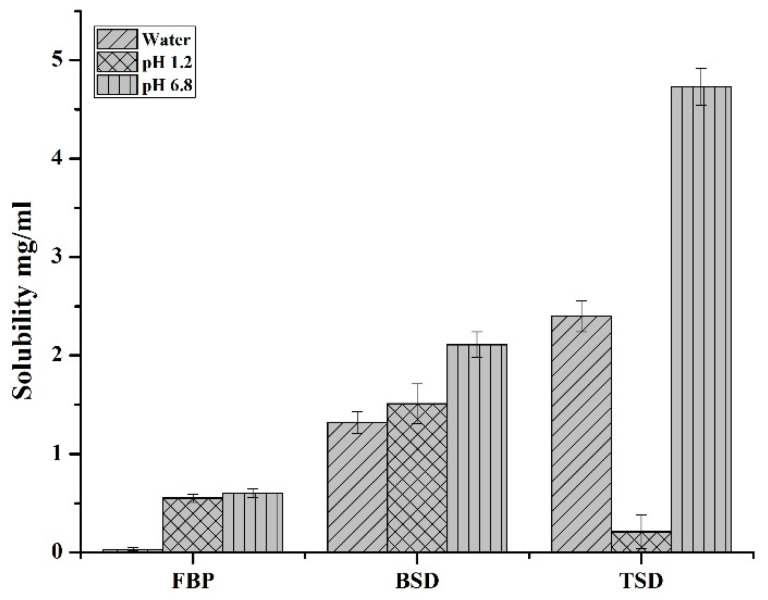
Solubility of FBP, BSD and TSD in water, pH 1.2 and pH 6.8.

**Figure 3:**
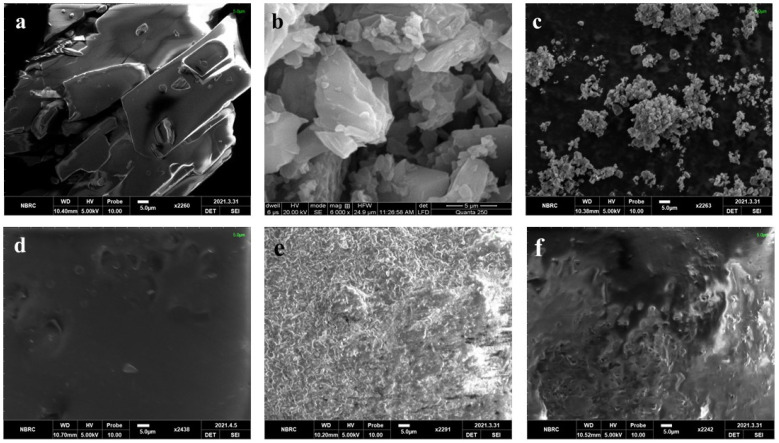
SEM of (**a**) FBP, (**b**) RHCl, (**c**) TSD, (**d**) S0, (**e**) S1 and (**f**) S2.

The solubility of pure FBP, the synthesized binary solid dispersion of FBP (FBP-BSD, coded as BSD) and the ternary solid dispersion of FBP (FBP-TSD, coded as TSD) were determined in water, pH 1.2 and pH 6.8 buffer. Figure 2 shows that at pH 1.2 FBP had slightly lower solubility than pH 6.8, which is due to its acidic nature. At low pH, FBP remained in nonionized form, showing slightly lower solubility. However, at a higher pH when the pH became greater than pKa, i.e., 4.22, FBP ionized to show slightly higher solubility. However, the difference is not that prominent as reported in the literature. This could be due to possibly be due to the use of USP hydrochloric acid buffer, which contains kosmotropic salt, i.e., potassium chloride, that helps in solubility enhancement, as reported in the literature [38,39]. Moreover, Figure 2 also clearly indicates that the solid dispersions augmented the solubility of pure FBP in water and at pH 6.8. Furthermore, the TSD showed more solubility (aqueous and at pH 6.8) as compared to BSD and pure FBP. TSD showed 24-times-increased solubility in water and 34 times at pH 6.8, while BSD showed 14-times-increased solubility in water and 15 times at pH 6.8 as compared to pure FBP. This significant improvement in TSD solubility could be due to the presence of Syloid^®^ 244FP in combination with poloxamer^®^ 188 as Syloid^®^ 244FP has a large surface area due to the presence of nano size pores, which enhances adsorption and inhibits drug crystallization, whereas poloxamer^®^ 188 improves the wettability of the hydrophobic drugs and also provides a synergistic effect with Syloid^®^ 244FP to augment the solubility of the drug [24]. At acidic pH, BSD showed slightly decreased solubility as compared to pure FBP. But at pH 1.2, with the addition of poloxamer^®^ 188 in TSD, a decrease in solubility was observed, with solubility levels of 1.78 mg/mL, 1.51 mg/mL and 0.21 mg/mL for pure FBP, BSD and TSD, respectively. These results gave us a clue that the addition of poloxamer^®^ 188 in TSD can impart a gastroprotective effect. Based on solubility, TSD was selected over BSD for further analysis and loading in composite ODFs. The powder density and compressibility values (i.e., bulk density 0.2098 g/mL, tapped density 0.221 g/mL, Carr’s index 5.06%, Hausner’s ratio 1.05 and angle of repose 20.3°) demonstrated that TSD powder had excellent flow and compressibility properties. It is reported that lower compressibility values are associated with high amorphous morphology [15]. In the SEM image, TSD appeared as aggregates of irregular shaped particles. Further, it revealed that FBP was loaded in amorphous form, as the drug crystals were not observed (Figure 3c), contrary to pure FBP (Figure 3a). The percentage drug content of FBP in TSD was 99.06% ± 1.65%, which is within the range according to USP27 (85–115%).

### 3.2. Composite ODF Properties

Composite ODFs were developed through solvent casting to produce the ternary solid dispersion of FBP (TSD) alone (S1) and in combination with plain RHCl (S2)-loaded ODFs with high reproducibility. The method is very simple, economical and does not require an organic solvent [14]. The composition of the film matrix was selected on the basis of our previous study [40]. The reproducibility of composite ODFs (S1 and S2) was assessed by measuring the percentage drug content and physical and mechanical parameters. The percentage drug content of FBP in S1 and S2 was 95.37 ± 2.22% and 95.22 ± 3.93%, respectively, while the percentage drug content of RHCl in S2 was 98.29 ± 1.05%.

### 3.3. Physical Parameters of Composite ODFs

#### 3.3.1. Thickness

Thickness is a physical parameter of the film that indicates the uniformity of the film. The film should be of optimum thickness as very thin films may easily rupture when they are peeled from the casting Petri dish, while thick films are reported to disintegrate slowly [22]. The thicknesses of various developed formulations are mentioned in Table 2. The thickness of formulations was within permissible limits, i.e., 50–200 µm as described by Lai, Fang [41].

#### 3.3.2. In Vitro Disintegration Time (DT)

In vitro disintegration testing demonstrated that both formulations, S1 and S2, disintegrated quickly, i.e., within 15 s, as stated in Table 2, which is suitable for an oral fast-disintegrating delivery system. The recommended DT of fast disintegrating tablets is 30 s or less, as per CDER guidelines, and is equally applicable for fast-disintegrating oral films [42]. The decrease in the DT of S1 and S2 films as compared to the S0 film is possibly due to the incorporation of solid dispersion in the HPMC matrix of the ODF, which causes discontinuation within the polymer lattice [43,44]. Similar results were stated by Łyszczarz, Hofmanová [10], who also showed a favorable impact of solid dispersion on the DT of the ODF. They reported that ODF containing aripiprazole–poloxamer^®^ 407 solid dispersion disintegrated faster (DT ˂ 30 s) than the blank film. Furthermore, the DT of S2 was slightly lower than S1, after the encapsulation of RHCl. This is possibly due to the presence of freely water-soluble RHCl, which further enhances the contact of the film with media and thus it disintegrates faster. For ODFs produced with two different grades of HPMC, Panraksa, Udomsom [45] achieved an average DT of 6 s. In a study conducted by Bodini, Guimarães [46], an HPMC-based film showed a DT of 33.4 s and starch-based film showed a DT of 43.7 s.

### 3.4. Mechanical Parameters of Composite ODFs

#### 3.4.1. Folding Endurance

Folding endurance gives an idea about the flexibility and brittleness of the films. The synthesized formulations (S0, S1, and S2) had folding endurance values greater than 300, as stated in Table 2. According to the literature, a film with a folding endurance of 300 or >300 exhibited excellent flexibility [47]. Here, the film-forming polymer, i.e., HPMC E5, imparts strength to the film, while the plasticizer, i.e., PG, provides flexibility to the ODF, as observed previously [48]. The optimum flexibility of films ensures their integrity until they reach the hands of patients.

#### 3.4.2. Tensile Strength

An optimal film should have optimal tensile strength to endure the force or stress during packaging and transportation without any damages [45]. Moreover, too rigid a film gives a bad mouthfeel [49]. The results demonstrated that the tensile strength of developed films were in accordance with the quality standard of ODF mentioned by Brniak, Maślak [48], i.e., below 10 N/mm^2^ (Table 2). The decrease in the tensile strength of S1 and S2 films as compared to the S0 film was possibly due to the presence of solid dispersion. It is assumed that the incorporation of ternary solid dispersion increases the discontinuations in the film matrix. These results are concordant with the previous findings [50], where increasing the concentration of micropellets in films reduced the mechanical properties by influencing the continuity of the polymer lattice. Łyszczarz, Hofmanová [10] showed that ODF containing solid dispersion had a somewhat decreased tensile strength as compared to the blank film. In another study, ODF containing prednisolone MPs presented similar results. The presence of MPs in ODF slightly lowered the tensile strength as compared to that of the blank film [48].

### 3.5. Solid State Characterization of TSD and Composite ODF

#### 3.5.1. SEM

The surface morphology of the pure drug and formulations were observed by SEM. Figure 3 illustrates the surface morphology of the pure drug (FBP and RHCl) and formulations (TSD, S0, S1, and S2). The SEM micrographs show crystals of FBP and RHCl that depict their crystalline nature (Figure 3a,b). SEM micrographs of TSD, S1 and S2 reflect amorphous characteristics due to the absence of drug crystals. Pradhan, Tran [5] reported that in developed FBP solid dispersion, FBP crystals were transformed into an amorphous state. SEM micrographs also revealed that solid dispersions are successfully embedded and well dispersed in composite films (S1 and S2). The surface of the blank was smoother but became rough upon incorporation of solid dispersion (S1). Furthermore, after RHCl loading, film (S2) became smoother, which indicates the successful incorporation of RHCl.

#### 3.5.2. FTIR

**Figure 4:**
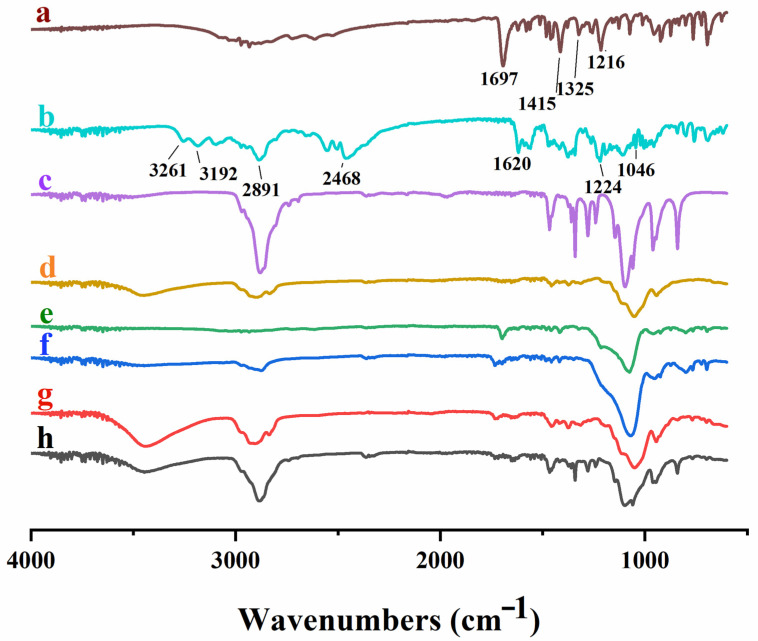
FTIR spectra of (**a**) FBP, (**b**) RHCl, (**c**) poloxamer^®^ 188, (**d**) HPMC E5, (**e**) Syloid^®^ 244FP, (**f**) TSD, (**g**) S1 and (**h**) S2.

FTIR analysis was used to confirm the chemical structure of polymers and to recognize any potential intermolecular interactions between functional groups of active ingredients and polymeric carriers. Figure 4 shows the FTIR spectrum of pure drugs (FBP and RHCl), polymers (HPMC E5, poloxamer^®^ 188 and Syloid^®^ 244FP) and developed formulations (TSD, S1 and S2). The spectrum of pure FBP presented a characteristic band between 3500 and 2500 cm^−1^, which was assigned to the stretching of the –OH of the hydroxyl group (3500–3000 cm^−1^) and –CH of the methyl group (3000–2500 cm^−1^). The distinct peaks at 1216 cm^−1^, 1325 cm^−1^, 1415 cm^−1^ and 1697 cm^−1^ were attributed to C–F stretching, –CH of the methyl group, –OH bending and the carbonyl group (C=O), respectively (Figure 4a). Kawadkar and Chauhan [51] and Liw, Teoh [52] reported similar peaks of FBP. The pure RHCl spectrum (Figure 4b) displayed characteristics peaks of C=N (nitronic acid) at 1619 cm^−1^, NO_2_ (nitro group) at 1224 cm^−1^, NH (CH_3_)_2_ (dimethylamine group) at 2468 cm^−1^ and two distinct peaks of primary amide group (NH) at 3192 and 3261 cm^−1^. A strong peak was observed at 1046 cm^−1^, which depicts the crystalline form II of RHCl. Gaitano, Calvo [53] and Chieng, Aaltonen [54] reported similar RHCl spectral vibrations. Poloxamer^®^ 188 demonstrated absorption peaks of C–O (ether) stretching at 1097 cm^−1^, C–O–C stretching at 1243 cm^−1^ and 1288 cm^−1^, –OH bending at 1343 cm^−1^ and 1464 cm^−1^ and aliphatic –CH stretching at 2886 cm^−1^ (Figure 4c) and are in agreement with previous results [21,55]. Principle absorption peaks of HPMC E5 were found at the wavenumber of 1055 cm^−1^ due to C–O stretching; 1375 cm^−1^ represented –OH group vibration, 1457 cm^−1^ presented a –CH_2_ group, and 2908.6 cm^−1^ indicated the stretching of –CH and 3449.5 cm^−1^ of –OH stretching (Figure 4d) [56], whereas Syloid^®^ 244FP showed its distinctive IR peaks at the wavenumbers of 1079 cm^−1^ and 1418 cm^−1^, which are attributed to Si–OH (silanol group) stretching and –OH bending, respectively (Figure 4e) [57]. In the IR spectrum of TSD, S1 and S2 (Figure 4f–h), the characteristics peaks of FBP and RHCl were weaker and hardly observed in their respective formulations due to the formation of hydrogen bonding between drug and carrier polymers. The other reason is the overlapping of peaks of drug and carrier polymers. For instance, the peak 1415 cm^−1^ of FBP was shifted to 1418 cm^−1^, 1421 cm^−1^ and 1417 cm^−1^ in TSD, S1 and S2, respectively, and it was attributed to overlapping with the silanol group peak of Syloid^®^ 244FP, and thus these peaks were less intense. Moreover, the peak at 1046 cm^−1^ of RHCl in S2 was shifted to a higher IR band, i.e., 1061 cm^−1^, due to overlapping with peak 1055 cm^−1^ of the C–O group of HPMC E5 [40]. The other reason could be the formation of strong –H bonding with film-forming polymer HPMC E5 [58]. The IR results confirmed that the pure drugs with reference to their formulations showed no obvious drug–polymer interactions. All formulations (TSD, S1 and S2) showed characteristic peaks of FBP and RHCl without any major shifting in their respective formulations, but these were weaker as compared to the pure drug. Similar results are reported for albedazole–PEG6000–poloxamer^®^ 188 solid dispersions (ABZ-SD), where the characteristic peak of albendazole was hardly observed in the IR spectrum of ABZ-SD, while the characteristic peaks of carriers were clear [23].

#### 3.5.3. XRD

**Figure 5:**
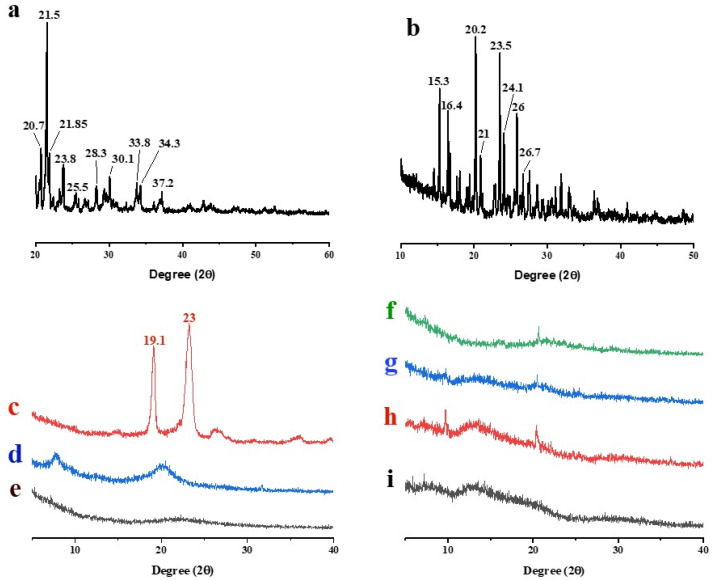
XRD of (**a**) FBP, (**b**) RHCl, (**c**) poloxamer^®^ 188, (**d**) HPMC, (**e**) Syloid^®^ 244FP, (**f**) TSD, (**g**) S0, (**h**) S1 and (**i**) S2.

The crystallinity or amorphicity of drugs, polymers and synthesized formulations were assessed by XRD. The XRD pattern of pure FBP exhibited several intense peaks between 20.7° and 30.1°, confirming its crystalline nature (Figure 5a) as reported in the literature [59,60]. The pure RHCl also depicted the typical crystalline diffraction peaks between 15.3° and 26° (Figure 5b), which were in agreement with previously published data by Yamamoto, Takeda [61]. Poloxamer^®^ 188 showed diffraction peaks at 19.1° and 23° (Figure 5c) [29,62], while HPMC E5 and Syloid^®^ 244FP (Figure 5d,e) did not exhibit characteristics peaks. TSD, S1 and S2 showed halo amorphous patterns (Figure 5f,h,i) that revealed the dispersion of drugs (FBP and RHCl) at the molecular level in their respective formulations. Zhang, Sun [24] developed a solid dispersion of cilostazol with Syloid^®^ 244FP and Kolliphor^®^ 188 and their XRD diffraction patterns revealed an amorphous nature. Similarly, XRD studies reported by Yeo, An [15] suggested that aprepitant was amorphously dispersed in Syloid^®^ 244FP-based dispersion.

### 3.6. In Vitro Drug Release of TSD and Composite ODFs

**Figure 6:**
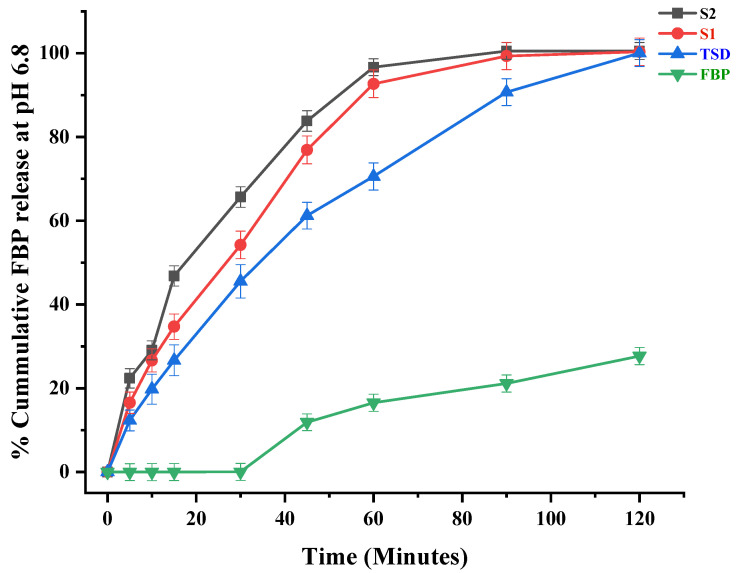
In vitro release profile of FBP at pH 6.8. Error bar represents mean ± SD (*n* = 3).

**Figure 7:**
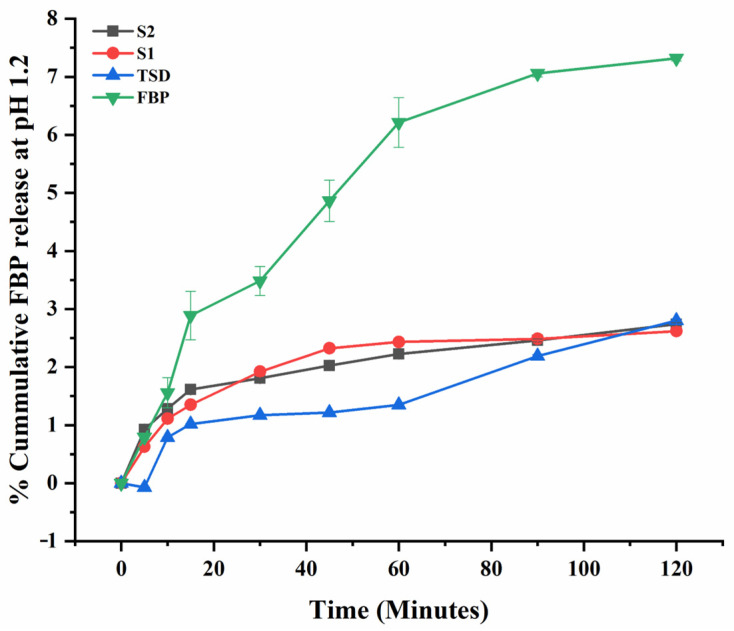
In vitro release profile of FBP at pH 1.2. Error bar represents mean ± SD (*n* = 3).

**Figure 8:**
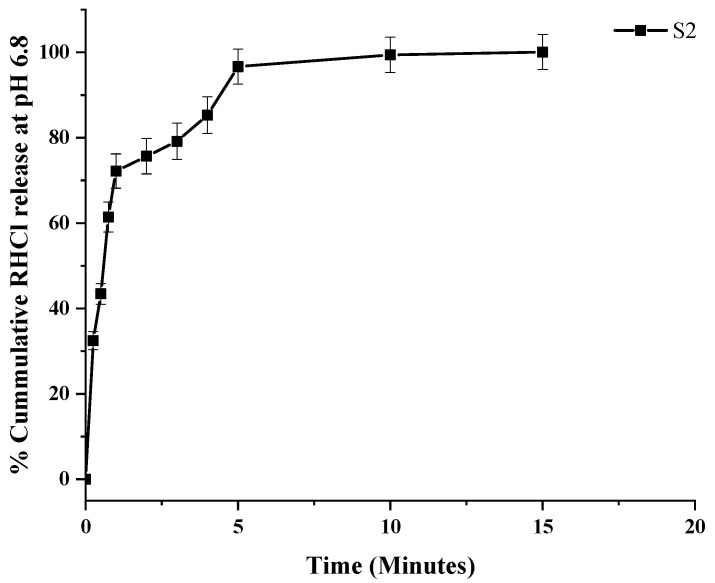
In vitro release profile of RHCl at pH 6.8. Error bar represents mean ± SD (*n* = 3).

The in vitro release profile of FBP at pH 6.8 and pH 1.2 and RHCl at pH 6.8 are graphically presented in Figure 6, Figure 7 and Figure 8, respectively. The results showed that the percentages of FBP released at pH 6.8 during the first 30 min from TSD, S1 and S2 were 45.53%, 54.22% and 65.66%, respectively (Figure 6). In contrast, almost negligible FBP was released at pH 1.2 from TSD, S1 and S2 (Figure 7). The results reveal that solid dispersion has improved the release profile of FBP at pH 6.8 owing to a large pore volume and large pore diameter of Syloid^®^ 244FP. Here, the nano-sized pore structure of Syloid^®^ 244FP inhibits drug nucleation. Furthermore, due to the presence of a large number of silyl hydroxyl groups on the surface of Syloid^®^ 244FP, it develops hydrogen bonding with the drug and inhibits crystallization to confine the drug in amorphous form in silica particles, while poloxamer^®^ 188 with Syloid^®^ 244FP imparts synergistic effects as described by Zhang, Sun [24] due to its surface active nature and micellar solubilization power. Moreover, poloxamer^®^ 188 coating also inhibits the release of the drug at pH 1.2 (Figure 7) and significantly improves intestinal absorption [19]. Thus, it ultimately prevents FBP-induced gastric irritation [18]. Composite ODFs (S1 and S2) exhibited a relatively faster release rate as compared to TSD. This was attributed to (1) the availability of higher surface area for wetting of TSD in ODF, (2) the presence of HPMC, superdisintegrant and plasticizer in composite ODF can act as a dissolution enhancer. Furthermore, S2 ODF showed a significantly more rapid release of FBP as compared to S1 that may be due to the presence of hydrophilic RHCl, as it was reported that RHCl enhanced the solubility and bioavailability of the hydrophobic diclofenac [53]. Similar results were observed by Kasai, Shiono [63], where the solubility and bioavailability of the hydrophobic diclofenac was enhanced by the ion-paired complex formation with hydrophilic cimetidine.

In the dual drug-loaded composite ODF (S2), the cumulative % release of RHCl at pH 6.8 was 96.66% within 5 min (Figure 8). Previously, a fast-disintegrating oral film reported by Satyanarayana and Keshavarao [64] showed a 90% release of anastrozole within 4 min from HPMC E5-based ODF, while another study showed 100% drug release within 6 min from HPMC-based ODF-containing donepezil [26].

### 3.7. In Vivo Study

#### 3.7.1. Assay of Analgesic Activity

**Figure 9:**
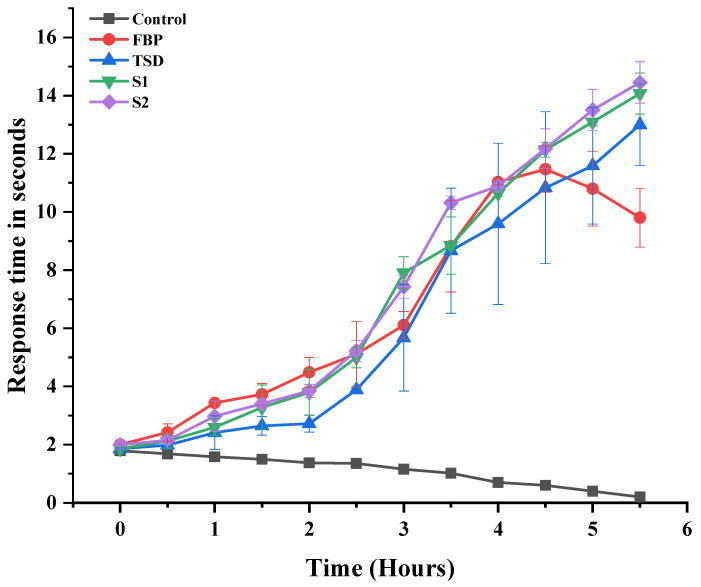
Analgesic activity of pure FBP and synthesized formulations (TSD, S1 and S2). Error bar represents mean ± SD (*n* = 3).

The ability of developed formulations (TSD, S1 and S2) to enhance the analgesic effect was assessed and compared with controlled (non-treated) and pure FBP (Figure 9). The in vivo analgesia effect was checked from 0.5 to 5.5 h by the hot plate method. The latency time to the thermal stimuli of the controlled group gradually decreased as it was more vulnerable to heat. The pure FBP showed the maximum latency time at the 4th h (11.025 ± 0.092 s); afterwards, the time gradually decreased (9.8 ± 1.01 s at 5.5 h). This short analgesic effect was attributed to the short half-life and poor solubility profile of FBP, whereas the latency time of the synthesized formulations TSD, S1 and S2 was significantly prolonged when compared to pure FBP. The maximum latency times for TSD, S1 and S2 were 13 ± 1.4, 14.07 ± 0.71 and 14.45 ± 0.707 s at 5.5 h, respectively. These results suggest that the analgesic activity of the FBP was increased after incorporation in solid dispersions and composite ODFs. The improvement in analgesic effect was attributed to mesoporous silica and poloxamer^®^ 188 carriers that enhanced the solubility and bioavailability of the FBP [15,55].

#### 3.7.2. In Vivo Anti-Inflammatory Activity

**Figure 10:**
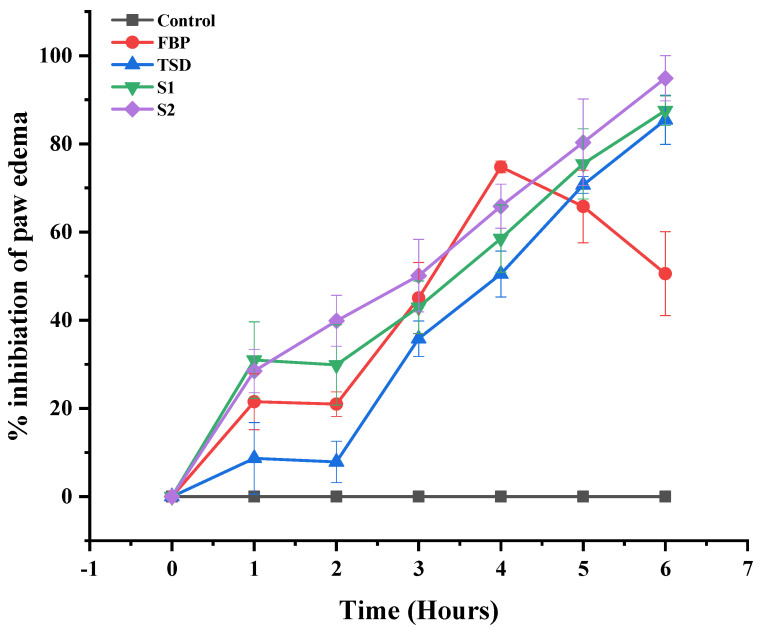
Percentage inhibition of paw edema after the administration of pure FBP and synthesized formulations (TSD, S1 and S2). Error bar represents mean ± SD (*n* = 3).

The carrageenan-induced rat paw edema model was used to assess and compare the in vivo anti-inflammatory efficacy of the developed formulations (TSD, S1 and S2) with the control (non-treated) and pure FBP treated groups. In the control group, after carrageenan injection, the paw edema showed zero inhibition (Figure 10). The pure FBP inhibited the development of edema up to 4 h (74.766% ± 1.32); afterwards, the percentage of inhibition decreased. In the case of TSD, S1 and S2, the percentage inhibition of the edema increased gradually up to 6 h, as shown in Figure 10. Moreover, the percentage inhibition was higher at all time points with all formulations when compared with pure FBP. Thus, these findings manifested that the loading of FBP in TSD and composite ODFs did not alter the release pattern of FBP but improved the therapeutic efficacy. Among the synthesized formulations, S2 showed the maximum percentage inhibition (94.996% ± 5.12) of paw edema, and it was attributed to the presence of hydrophilic RHCl, which improves the solubility as well as bioavailability of FBP. Secondly, RHCl inhibits histamine release, which provides the antiedematous effect [65]. Hence, this indicates the synergistic anti-inflammatory effect of RHCl in S2 formulation with FBP.

##### Detection of Pro-Inflammatory Cytokines

**Figure 11:**
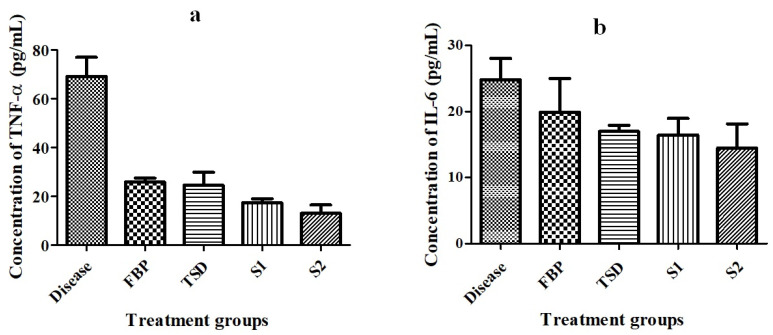
Rat blood serum level of (**a**) TNF-α and (**b**) IL-6 in carrageenan-induced paw edema model. Error bar represents mean ± SD (*n* = 3).

TNF-α and IL-6 are pro-inflammatory cytokines, which play fundamental roles in the initiating, maintaining and resolving of inflammation [66]. Therefore, levels of TNF-α and IL-6 were assessed in the blood serum of animals with paw edema induced by carrageenan. After 24 h of treatment with pure FBP and optimized formulations (TSD, S1, and S2), serum levels of pro-inflammatory cytokines were quantified by ELISA. The levels of TNF-α and IL-6 were noticeably increased in the diseased (control) group (41.607 ± 8.9 pg/mL, 14.96 ± 7.05 pg/mL, respectively) as compared to treated groups (pure FBP: 15.565 ± 4.2 pg/mL, 12.002 ± 6.51 pg/mL; TSD: 14.76 ± 4.82 pg/mL, 10.24 ± 9.2; S1: 10.459 ± 9.6 pg/mL, 9.91 ± 9.4 pg/mL; and S2: 7.855 ± 8.2 pg/mL, 8.73 ± 4 pg/mL, respectively) (Figure 11). Özdoğan, Akca [18] reported that the gel formulation of atorvastatin solid dispersion prepared with Pluronic F-68 significantly decreased the IL-6 level as compared to PEG 6000-based solid dispersion formulation. The animals treated with S2 displayed remarkably reduced TNF-α and IL-6 levels in contrast to the other treated groups. This might be due to the presence of H_2_ receptor antagonist (hydrophilic RHCl). Our results are supported by a previous study by Li, Huang [67] where cimetidine (H_2_ receptor antagonist)-treated group showed a significantly decreased level of TNF-α and IL-6 when compared to the diseased (ulcer) group. So, our findings suggest that S2 formulation exhibited superior efficacy to that of pure FBP and other formulations (TSD and S1). Li, Hu [68] reported that the hydrophilic drug (oxymatrine) enhanced the solubility and bioavailability of the hydrophobic compound (apigenin) in their co-amorphous mixture when compared to pure apigenin. Moreover, the level of inflammatory factors (TNF-α, IL-6, MCP-1 and COX-2) was significantly reduced by the presence of the hydrophilic drug in the co-amorphous mixture of apigenin. These results are in agreement with our results.

#### 3.7.3. Evaluation of Gastroprotective Activity

##### Gastric Lesion Index (GLI)

**Figure 12:**
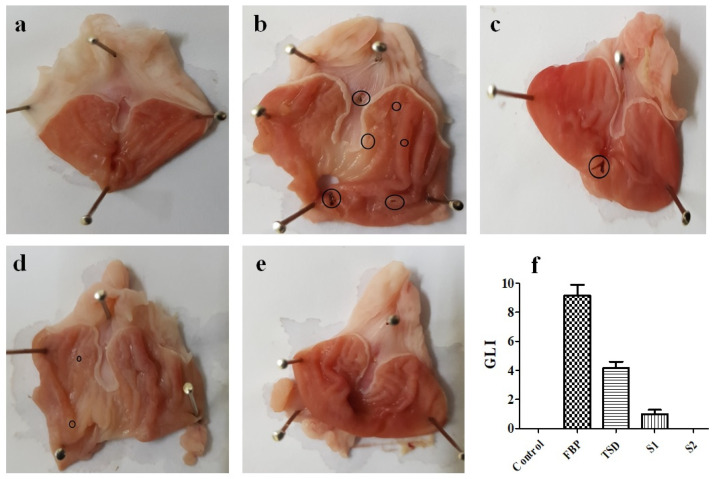
Photo-macrographs of rat stomach: (**a**) control group, (**b**) pure FBP treated group, (**c**) TSD treated group, (**d**) S1 treated group, (**e**) S2 treated group and (**f**) gastric lesion index. Error bar represents mean ± SD (*n* = 3). Black circles indicate ulcers.

The stomach morphology of rats was observed to examine the gastroprotective effect of developed formulations (TSD, S1, and S2). The macroscopic stomach morphology and gastric lesion index (GLI) of each group are shown in Figure 12. The stomach morphology of the FBP-treated group (Figure 12b) revealed a higher ulcer index, 9.166 ± 1.258 (Figure 12f). For rats in the control group, the macroscopic morphology remained normal (Figure 12a). The average score of gastric lesions was in order of pure FBP (9.166 ± 1.258) > TSD (4.166 ± 0.764) > S1 (1 ± 0.5) > S2 (0 ± 0) and control (0). The findings suggest that developed solid dispersion-based formulations were less toxic than pure FBP, as the presence of poloxamer^®^ 188 provides a gastroprotective effect [62]. Moreover, the macroscopic morphology as shown in Figure 12 clearly demonstrated that composite oral films (S1 and S2) markedly reduced the ulcer indices. Furthermore, rats receiving S2 formulation loaded with FBP-TSD and RHCl presented normal macroscopic morphology (Figure 12e). Here, RHCl along with poloxamer^®^ 188 could have acted synergistically to provide gastric mucosal protection.

##### Histopathological Analysis

**Figure 13:**
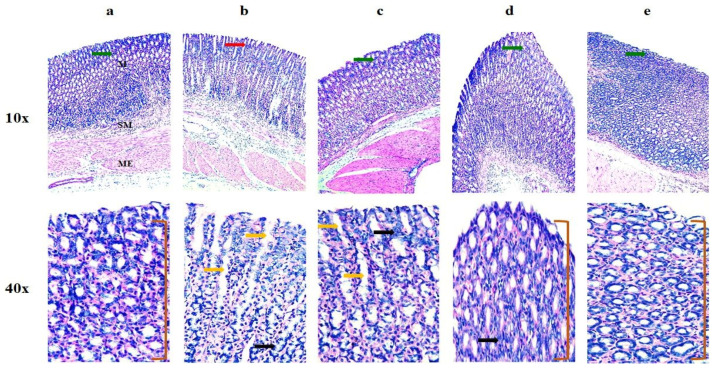
Histological images of stomach taken at various resolution in different groups. (**a**) Control group, (**b**) pure FBP-treated group, (**c**) TSD-treated group, (**d**) S1-treated group, and (**e**) S2-treated group. “M” is mucosa, “SM” is submucosa and “ME” is muscularis externa. Green arrows show intact mucosa. Red arrow shows degeneration of epithelium. Yellow arrows show edema. Black arrows show inflammatory infiltration. Bracket shows normal mucosal folds.

The histopathological examination of the gastric mucosa of the control group showed unchanged gastric architecture (Figure 13a). However, the FBP-exposed stomach showed an extensive disruption of mucosal layers with necrotic lesions, as evident from the inflammatory infiltration into the deeper layer of the mucosa and submucosa (Figure 13b). The TSD-treated group showed mild mucosal disruption and necrotic lesions with inflammatory infiltration into the deep mucosal layer (Figure 13c). The gastric mucosa of the S1-treated group showed almost normal architecture, without any significant pathology (Figure 13d) but with a 0.5 GLI score. Likewise, the S2-treated group (Figure 13e) showed a healthy mucosal lining, free from any significant pathology and degenerative changes, with no difference in the GLI score (0) from the control (Figure 12f). Here, S2 protected the stomach from FBP-induced ulcers. Thus, S2 is deemed to be non-toxic, safe and biocompatible following oral administration.

## 4. Conclusions

The co-administration of NSAID and the H_2_ receptor antagonist was successfully achieved by composite ODFs (S1 and S2) prepared by the casting method with HPMC E5 as the polymer and PG as plasticizer. All formulations were comprehensively characterized. The BSD of FBP was formulated with Syloid^®^ 244FP, which enhanced the solubility of FBP at pH 6.8, i.e., by about 15 folds, while the TSD of FBP was formulated with Syloid^®^ 244FP and poloxamer^®^ 188 that markedly enhanced FBP solubility over 34 folds. Based on solubility, the TSD was further characterized and loaded in composite films alone (S1) and in combination with RHCl (S2). The SEM and XRD analyses indicated that the drug was entrapped in amorphous form in TSD, which inhibited drug crystallization. Likewise, SEM and XRD analyses of ODFs confirmed that the drug remains in an amorphous state. The in vitro studies revealed the maximum release of drug at pH 6.8. Moreover, the in vivo studies revealed that formulation S2 had higher therapeutic efficacy as compared to FBP and other formulations (TSD and S1). Overall, the results evidentially demonstrated that composite ODF could be an effective carrier system for co-loading to enhance bioavailability, and to bypass the gastrointestinal and hepatic metabolic system. Moreover, it is suitable for combination therapy along with a gastroprotective effect in geriatric patients suffering from RA.

## Figures and Tables

**Table 1:** Composition of composite ODFs.

Code	HPMC E5	PG	Pearlitol Flash^®^	FBP TSD	RHCl	Dis. Water
S0	0.3 g	0.03 g	0.03 g			10 mL
S1	0.3 g	0.03 g	0.03 g	0.15 g		10 mL
S2	0.3 g	0.03 g	0.03 g	0.15 g	0.075 g	10 mL

**Table 2:** Physical and mechanical properties of composite ODFs.

Code	Thickness (µm)	Disintegration Time (s)	Folding Endurance	Tensile Strength (N/mm^2^)
S0	35 ± 0.58	19.51 ± 0.21	>300	8.3 ± 1.4
S1	62 ± 0.61	15.78 ± 0.94	>300	6.17 ± 0.31
S2	66.4 ± 0.67	15.02 ± 0.9	>300	5.3 ± 0.4

## Data Availability

Most of the data are presented in the article. However, the raw or processed data that were required to reproduce these findings cannot be shared at this time due to technical or time limitations.

## Abbreviations

API, Active pharmaceutical ingredient; BCS, Biopharmaceutical classification system; BSD, Binary solid dispersion; CDER, Center for drug evaluation and research; Ci, Compressibility index; CMC, Carboxy methyl cellulose; COX-2, Cyclooxygenase 2; DCM, Dichloromethane; DSC, Diffraction scanning calorimetry; DT, Disintegration time; ELISA, Enzyme-linked immunosorbent assay; FBP, Flurbiprofen; FTIR, Fourier transform infrared; GI, Gastrointestinal; GLI, Gastric lesion index; HPMC E5, Hydroxypropyl methyl cellulose E5; IL-6, Interleukin 6; M, Mucosa; ME, Muscularis externa; MCP-1, Monocyte chemoattractant protein 1; MPA, Maximum possible analgesia; NSAIDs, Non-steroidal anti-inflammatory drugs; ODFs, Oral fast disintegrating film; PF, Pearlitol Flash^®^; PG, Propylene glycol; RA, Rheumatoid arthritis; RHCl, Ranitidine hydrochloride; SD, Solid dispersion; SEM, Scanning electron microscope; SM, Submucosa; TNF-α, Tumor necrosis factor alpha, TSD, Ternary solid dispersion; XRD, X-ray diffraction.
